# A gene-based radiation hybrid map of the gilthead sea bream *Sparus aurata *refines and exploits conserved synteny with *Tetraodon nigroviridis*

**DOI:** 10.1186/1471-2164-8-44

**Published:** 2007-02-07

**Authors:** Elena Sarropoulou, Rafaella Franch, Bruno Louro, Deborah M Power, Luca Bargelloni, Antonios Magoulas, Fabrice Senger, Matina Tsalavouta, Tomaso Patarnello, Francis Galibert, Georgios Kotoulas, Robert Geisler

**Affiliations:** 1 Institute of Marine Biology and Genetics, Hellenic Center for Marine Research, Crete, Greece; 2 MPI fuer Entwicklungsbiologie, Spemannstr.35/III D-72076 Tuebingen F.R. Germany; 3 Dipartimento di Sanità Pubblica, Patologia Comparata e Igiene Veterinaria, Università di Padova, Padova Italy; 4 Campus de Gambelas, 8000-810 Faro, Portugal; 5 Department of Biology, University of Padova, Padova, Italy; 6 CNRS UMR 6061 Génétique et Développement, Université de Rennes 1, Faculté de médecine, 2 avenue du Pr Léon Bernard, 35043 Rennes Cedex, France; 7 CNRS UMR6026, Interactions Cellulaires et Moléculaires, Université de Rennes 1, Campus de Beaulieu, 35042 Rennes Cedex, France; 8 Current address: School of Biology and Environmental Science, University College Dublin, Belfield, Dublin 4, Ireland

## Abstract

**Background:**

Comparative teleost studies are of great interest since they are important in aquaculture and in evolutionary issues. Comparing genomes of fully sequenced model fish species with those of farmed fish species through comparative mapping offers shortcuts for quantitative trait loci (QTL) detections and for studying genome evolution through the identification of regions of conserved synteny in teleosts. Here a comparative mapping study is presented by radiation hybrid (RH) mapping genes of the gilthead sea bream *Sparus aurata*, a non-model teleost fish of commercial and evolutionary interest, as it represents the worldwide distributed species-rich family of Sparidae.

**Results:**

An additional 74 microsatellite markers and 428 gene-based markers appropriate for comparative mapping studies were mapped on the existing RH map of *Sparus aurata*. The anchoring of the RH map to the genetic linkage map resulted in 24 groups matching the karyotype of *Sparus aurata*. Homologous sequences to *Tetraodon *were identified for 301 of the gene-based markers positioned on the RH map of *Sparus aurata*. Comparison between *Sparus aurata *RH groups and *Tetraodon *chromosomes (karyotype of *Tetraodon *consists of 21 chromosomes) in this study reveals an unambiguous one-to-one relationship suggesting that three *Tetraodon *chromosomes correspond to six *Sparus aurata *radiation hybrid groups. The exploitation of this conserved synteny relationship is furthermore demonstrated by in silico mapping of gilthead sea bream expressed sequence tags (EST) that give a significant similarity hit to *Tetraodon*.

**Conclusion:**

The addition of primarily gene-based markers increased substantially the density of the existing RH map and facilitated comparative analysis. The anchoring of this gene-based radiation hybrid map to the genome maps of model species broadened the pool of candidate genes that mainly control growth, disease resistance, sex determination and reversal, reproduction as well as environmental tolerance in this species, all traits of great importance for QTL mapping and marker assisted selection. Furthermore this comparative mapping approach will facilitate to give insights into chromosome evolution and into the genetic make up of the gilthead sea bream.

## Background

Fish species constitute an exceedingly diverse group representing roughly half of the extant vertebrate species. More than 95 % of all living fish species are represented by the ray-finned fishes (actinopterygians) of which more than 99.8 % are teleosts. Their high level of morphological, behavioral, and ecological diversity makes the study of teleosts of real importance in attempts to address and resolve evolutionary questions. Furthermore teleost studies are of great intrinsic interest since they are economically important in both fisheries and aquaculture. In recent years due to the efforts made in genome studies of many fish species, especially of model fish species like zebrafish and *Tetraodon*, genomic information of vertebrates has shown a substantial increase and comparative genomics studies have become a very important method for studying genome evolution in teleosts and vertebrates in general [1] as well as for the identification of regions of conserved synteny (e.g. for review [2]).

The opportunity of comparing genomes of model fish species with those of farmed fish species can facilitate functional studies, such as the detection of candidate genes and regions for the identification of qualitative and quantitative trait loci (QTLs). Furthermore comparative genomics can improve on the time-consuming work of identifying genes affecting trait variability through QTL mapping by offering shortcuts and hypothesis-based approaches rather than random scan approaches. Nevertheless, this promising approach has until now been hampered by the limited number of genome projects because of the expensive technology involved. A powerful method that allows comparative genome analysis to be conducted by simple means constitutes comparative mapping, enabling comparison of syntenies and gene orders to be carried out [3-7]. Whereas for model fish species such as the zebrafish, *Tetraodon*, fugu and medaka, comparative mapping is a common practice, in non-model fish species of commercial as well as of evolutionary and ecological interest only a few studies have so far been published e.g. [8].

In contrast to studies concerning agricultural animals, maps of DNA markers and genes allowing QTL analysis are relatively rare for cultured fish species. However, linkage maps among aquaculture fish species are available for salmonid species [9,10], tilapia [11], channel catfish [12,13], Japanese flounder [14] and the common carp [15]. Among Mediterranean species linkage maps for *Sparus aurata *[16] and for another important marine aquaculture species, *Dicentrarchus labrax *[17] have recently been published. In addition to the genetic linkage map of the gilthead sea bream, a first generation of RH map has also been constructed [18]. Radiation hybrid mapping results in dense and reliable genome maps for comparative use, since, unlike linkage mapping, it is not dependent on polymorphism and permits easy mapping of genes and of neutral polymorphic markers.

In the present study comparative mapping is taken with the gilthead sea bream (*Sparus aurata*), a key species for large-scale Mediterranean aquaculture. The gilthead sea bream, a non-model fish species of commercial and evolutionary interest, is distributed in the Atlantic Ocean and the Mediterranean Sea [19,20] and represents the worldwide-distributed species rich family of Sparidae, within the Perciformes. Comparative mapping for the gilthead sea bream *Sparus aurata *is reported through a gene-based radiation hybrid map with 428 markers including candidate genes for QTL and 74 microsatellite markers integrated with the previously published map of [18].

Furthermore, the considerable potential of comparative mapping for transferring information from model species to non-model species is demonstrated by the exploitation of conserved synteny. This established syntenic relationship between sea bream and *Tetraodon *enables to virtually map on the RH map ESTs of gilthead sea bream that give a significant similarity hit to *Tetraodon*. The sea bream RH map facilitates the scanning for QTLs mainly controlling growth, disease resistance, sex determination and reversal, reproduction as well as environmental tolerance, all traits of great importance for aquaculture. It also contributes to the identification of regions of conserved synteny and thereby provides a resource for further comparative mapping analysis between fish species and pinpoints possible chromosomes splitting, chromosomes fusions and chromosomes rearrangements during evolution.

## Results

### RH mapping

**Figure 1:**
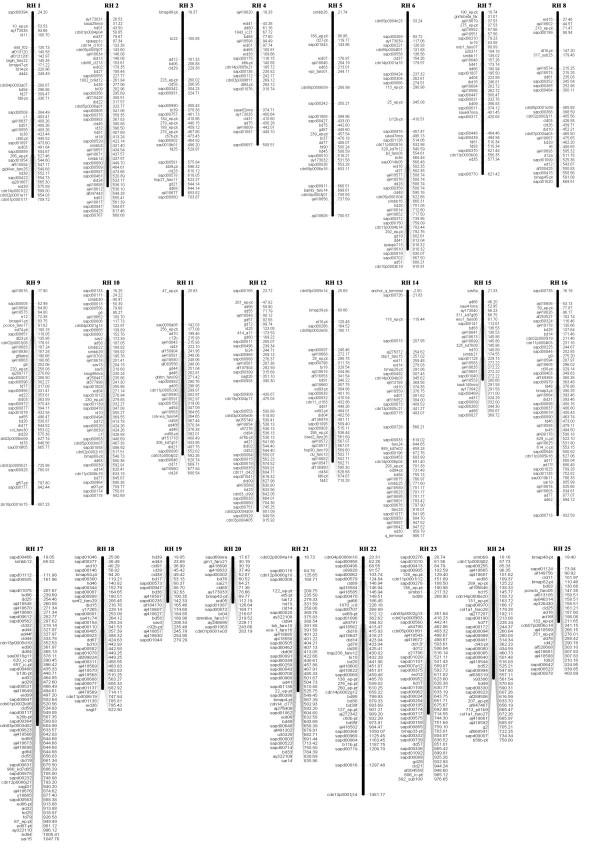
Radiation hybrid map of *Sparus aurata *consisting 25 radiation hybrid groups and 937 molecular markers. RH: Radiation hybrid group.

An additional 74 microsatellite markers and 428 ESTs were successfully positioned and integrated into the RH map produced by [18] (Figure 1, see Additional file 1). In total 25 RH groups were built from the newly mapped markers and from those mapped previously [18], resulting in a total number of 937 molecular markers on the *Sparus aurata *RH map. RH groups were renumbered compared to [18] where 28 RH groups were constructed. Since the number of chromosomes in this species is 24 [21,22], at least two of the current radiation hybrid groups must correspond to one chromosome. We anticipate that in future maps the smallest RH groups 19 and group 20, will be merged into one group as they correspond to the same genetic linkage group, and comparative mapping indicates that they also match the same chromosome in *Tetraodon *(see below). In this case the number of RH groups (24), that would result, correspond to the number of chromosomes in sea bream.

### Quality control

**Figure 2:**
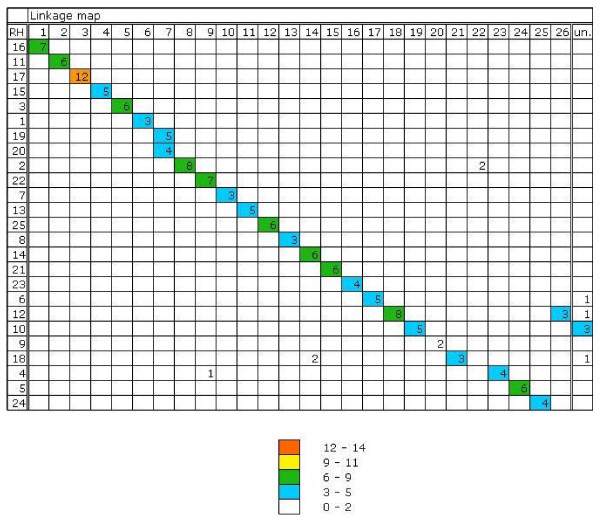
Matches between the *Sparus aurata *linkage map and radiation hybrid groups (RH groups are renumbered compared to [16] and [18]) shown in Oxford grid format, sorted by best matches. The number in each square is the number of matching genes. RH: radiation hybrid groups, un.: unassigned markers.

The reliability of the dataset was proved by mapping a set of genes and microsatellite markers (11) mapped in the first generation of RH map [18] (sequences coming from NCBI) again with new designed primers based on ESTs coming from cDNA libraries produced within the BRIDGEMAP project (see Additional file 2). Comparison with the genetic linkage map confirms the reliability of the obtained dataset. Twenty-six of the newly mapped microsatellite markers had previously been positioned on the genetic linkage map constructed by [16], and have been used to integrate the RH map and the genetic map. Comparison of the RH map and the linkage map shows that most markers found in one linkage group are also found in a single RH group, with the exception of eight markers from four linkage groups that were placed in a different RH group (Figure 2). For those new primer pairs were designed to confirm their position on the RH map. Furthermore, a set of markers (including markers already mapped by [18]) was genotyped twice resulting in the same vector scheme.

### Locus matching

The loci, successfully mapped on the sea bream RH map, were used to search for homology against the genome of two model species, *Tetraodon nigroviridis *and *Danio rerio*. The searches were performed by running BLAT [39] with a threshold score higher than 77, as well as BLAST with a threshold E-value <10 ^-4 ^and a minimum alignment length of more than 50 bp, both against the ENSEMBL database (v.38 – Apr2006) of these three species (see Additional files 3 and 4). Searches with BLAST and BLAT generally gave similar results. The BLAST search against *Tetraodon*, another Perciformes, resulted in 5% more positive hits than BLAT, while against Danio there were 19% more positive hits (Table 1). In general, BLAST searches resulted in a higher number of positive matches in all three species compared to BLAT, a result inherent in the algorithms employed, which should be taken into account when using these for homology searches between species.

### Comparative mapping

**Figure 3:**
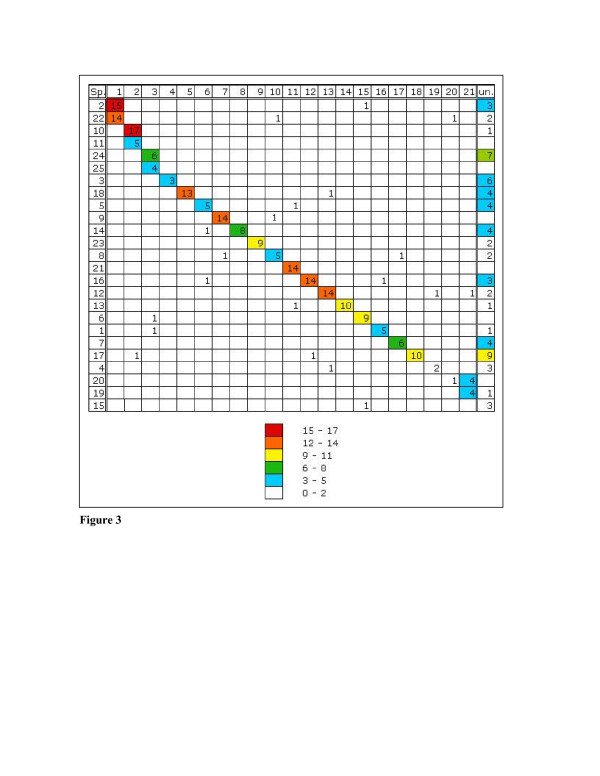
Oxford grid showing conservation of synteny between *Sparus aurata *and *Tetraodon nigroviridis*, sorted by best matches between *Sparus *radiation hybrid groups and *Tetraodon *chromosomes. The number in each square is the number of matching genes. Sp.: *Sparus*, un.: unordered random sequences.

Comparative mapping with all marker sequences available was performed using the BLAT web server with the *Tetraodon *genome and the *Danio rerio *genome for which an ordered map is available. Comparative mapping in *Tetraodon *resulted in the successful assignment of 301 *Sparus aurata *sequences to sequences of the *Tetraodon *genome. Of those 62 were assigned to unordered random sequences (Un_random). The remaining 239 sequences gave synteny groups covering all sea bream RH groups, with a mismatch rate of 8% (20 markers not found in synteny groups) (Figure 3).

Comparative mapping of *Sparus *against *Danio *with the BLAT web server gave only 90 hits, out of which 5 were not assigned to a chromosome (NA_random). Syntenic relationships between *Sparus aurata *and *Danio *were not as apparent as in *Tetraodon*.

## Discussion

The gilthead sea bream unlike the model organisms zebrafish and medaka, mostly used to study diseases and malfunctions, is a species of great commercial interest. Consequently, considerable information has been gathered on different aspects of its husbandry, physiology, biology and pathology, while a comprehensive genomic "tool box" has been created. The basis for sea bream genomics was recently established with the creation of a first generation linkage map [16] and radiation hybrid map [18]. The power of the RH map is significantly increased in the present study with the mapping of ESTs and this will be an important resource for future QTL detection and identification of functional units. Moreover, the present RH map represents a significant tool for comparative mapping as the sea bream belongs to the successful order of Perciformes which underwent an explosive radiation 50–70 million years ago.

### Comparison of the radiation hybrid map to the linkage map

**Figure 4:**
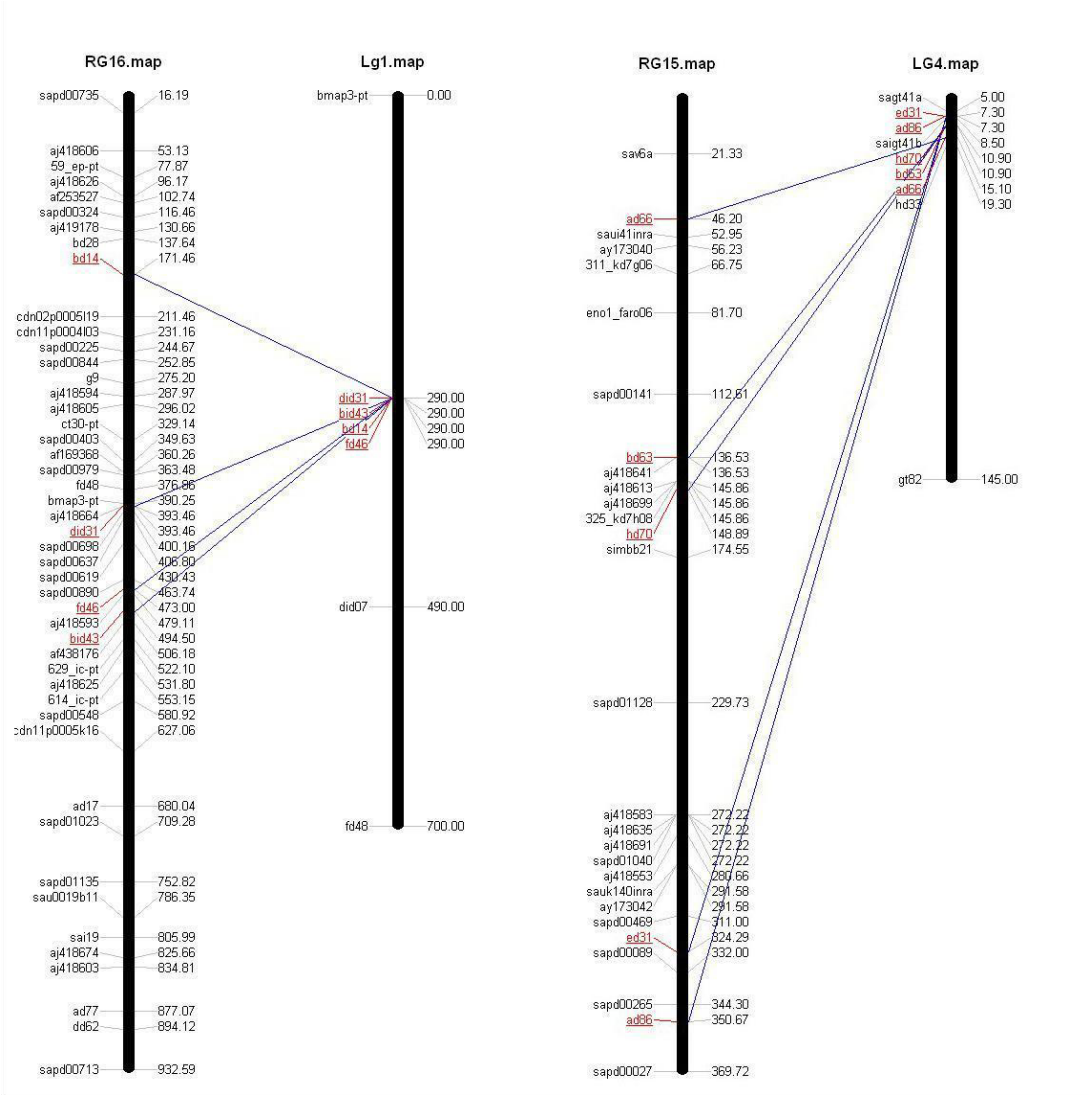
Comparison of *Sparus aurata *radiation hybrid group 16 with genetic linkage group 1 and radiation hybrid group 15 with genetic linkage group 4, according to data from [16].

In contrast to genetic linkage maps, radiation hybrid mapping allows the mapping of non-polymorphic molecular markers such as ESTs or genes. Markers are assigned based on their retention in specific members of the panel of cell lines. The current RH map gives a higher resolution of insufficiently resolved areas of the genetic map and allows recombination hot spots to be predicted (Figure 4). Twenty-six out of the additional 74 microsatellite markers newly mapped were also positioned on the genetic linkage map by [16] and can be used to anchor the genetic and the radiation hybrid map to each other. The discrepancy of eight markers (Bd 61, Dld 24, Bmap 54-PT, SaGT1, Ad 75, Hd23, G4 and Dld 09) between the two maps occurs as it is expected that some linkage groups will be modified with the addition of new markers. Linkage group 22 contains the two markers, Bd 61 and Dld24, mapping to the RH group 2 in this study. As already mentioned in [16] it is likely that linkage group 8 and linkage group 22, both corresponding to RH group 2, will merge into a single group. This is also the case for markers Hd23, G4 (myogenic factor) and Dld09 mapping to linkage group 26 which is merging together with linkage group 18 into one group (RH12) [16]. The marker Ad75 positioned on linkage group 9 and RH 4 is likely to belong to linkage group 23 as [16] could not position this marker in relation to the other markers on linkage group 9. Ad75 was reported by [18] as an independent group (RH 25 in [18]) together with AY173035, AJ418609 and Cld 31. All four were grouped to RH 4 (RH24 and RH25 of [18]) in this study. The linkage group 14 most likely breaks between Bmap 19-PT and Eid 11, as the distance between these two markers is large. Probably the first half on linkage group 14 including the two markers, SaGt1 and Bmap 54-PT is actually merging with linkage group 21 (linkage group 21 contains only four markers not positioned in a specific order) corresponding to RH group 18.

### Comparative mapping

**Figure 5:**
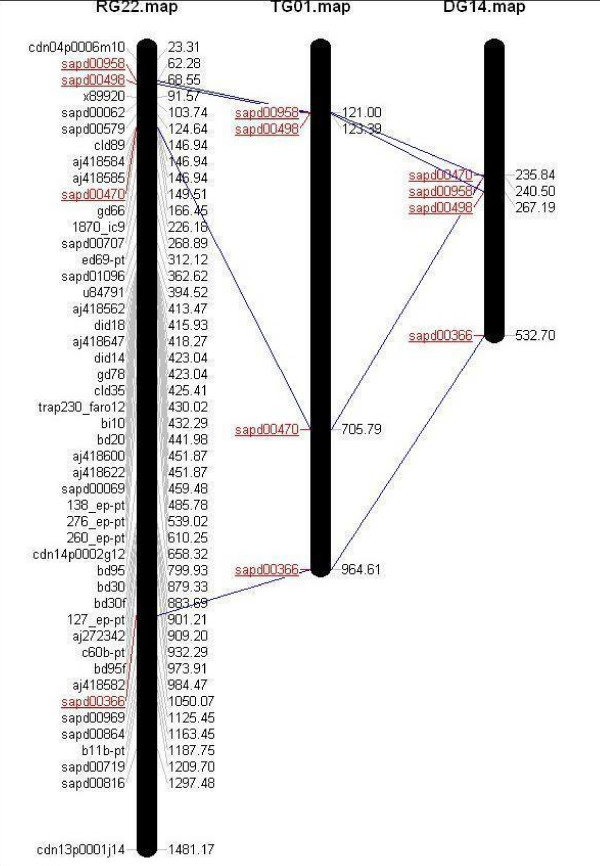
Comparison of *Sparus aurata *radiation hybrid group 22 with *Tetraodon *chromosome 1 and *Danio rerio *chromosome 14.

Previous studies using the sea bream genetic linkage map [16] and the first generation sea bream radiation hybrid map [18] gave evidence of conserved synteny. While the present analysis consolidates and extends the results of the first indication, synteny conservation between *Tetraodon nigroviridis *and *Sparus aurata *is demonstrated in greater detail by increasing the RH map coverage. For comparison with a more distantly related species, RH markers were also mapped to the *Danio rerio *genome (March 2006 zebrafish [*Danio rerio*] Zv6 assembly produced by The Wellcome Trust Sanger Institute in collaboration with the Max Planck Institute for Developmental Biology [Tuebingen, Germany], and the Netherlands Institute for Developmental Biology [Hubrecht Laboratory, Utrecht, The Netherlands]). Comparison between *Danio rerio *and *Sparus aurata *resulted only in small syntenic groups of up to 5 markers. Some of the markers are syntenic across all three species, such as four markers on chromosome 14 of *Danio*, chromosome 1 in *Tetraodon *and RH group 22 in sea bream (Figure 5). In parallel with BLAT, BLAST searches were also performed against the same databases. Though these gave slightly more hits, they were less successful in the detection of synteny groups (data not shown), which may be attributed to the fact that, among distantly related species, BLAST can detect more divergent or shorter alignments of uncertain homology. Reciprocal BLAST searching, frequently used to establish orthology, is currently not a valid option for sea bream due to the relatively small number of ESTs available. We therefore believe that the more stringent BLAT algorithm is the preferred method for comparative mapping in this study. For the following analysis we focused on *Tetraodon*, because it gave more BLAT hits than *Danio *due to its closer kinship while also providing an ordered map [1]. As information on conserved synteny of *Tetraodon *and *Danio *(as well as other model organism fish such as *Tilapia*) can be readily extracted from the genome databases, information from such model organisms can be utilized for *Sparus *research by way of the *Tetraodon *genome. Mapping of all RH markers to the *Tetraodon *genome showed that 16 of the 21 *Tetraodon *chromosomes could each be clearly matched with a single *Sparus *RH group (Figure 3). For these chromosomes 80 % of the matching markers are from only one *Sparus *RH group. Conversely, 22 of the 25 *Sparus *RH groups can be matched with a single *Tetraodon *chromosome, with 89 % of the matching markers hitting the same chromosome (excluding those markers that hit unordered random sequences). Only *Tetraodon *chromosome 20 was not clearly assigned to any *Sparus *RH group. This was because only two RH markers were be able to be mapped on *Tetraodon *chromosome 20 and those two matched to two different RH groups. From the 6806 EST of Marine Genomics which were produced randomly out of 14 different cDNA libraries, only 34 ESTs matched *Tetraodon *chromosome 20 (Table 2). Compared to the other chromosomes with matching EST markers from 93 to 398, the number of EST markers matching to chromosome 20 is surprisingly low, indicative perhaps of high variability or a low number of genes on this chromosome. Since *Sparus *RH group 15 did not match to a *Tetraodon *chromosome it may be postulated that RH group 15 may correspond to *Tetraodon *chromosome 20. Mapping of the 34 EST markers found to be located on chromosome 20 is underway. In general, there seems to be an indication for a one-to-one relationship between *Sparus *and *Tetraodon *chromosomes. Given that *Tetraodon *has 21 chromosomes, such a one-to-one accordance is obviously not to be expected for all chromosomes. Our data suggest that four *Tetraodon *chromosomes correspond to major portions of at least two *Sparus *radiation hybrid groups, namely *Tetraodon *Chr1 to *Sparus *RH2 and RH22, Chr2 to RH10 and RH11, Chr3 to RH24 and RH25, and Chr21 to RH19 and RG20 (the latter two RH groups may actually represent one *Sparus *chromosome, as noted above). The consecutively numbered RH groups in three of the cases are coincidental as the numbering of the RH groups is done randomly by the RH software. Interestingly, [23] proposed that *Tetraodon *chromosomes 1 and 2 (the two largest chromosomes) each correspond to two chromosomes of Danio rerio. However the authors also proposed a correspondence for Tetraodon chromosomes 7, 11, 12 and 13 with *Danio rerio *pairs of chromosomes, which according to our analysis corresponds to a single Sparus RH group. This may indicate that the duplication and/or rearrangement events affecting the four latter chromosomes occurred in the lineage leading to *Danio*, after its split with the linage leading to *Sparus *and *Tetraodon*.

**Figure 6:**
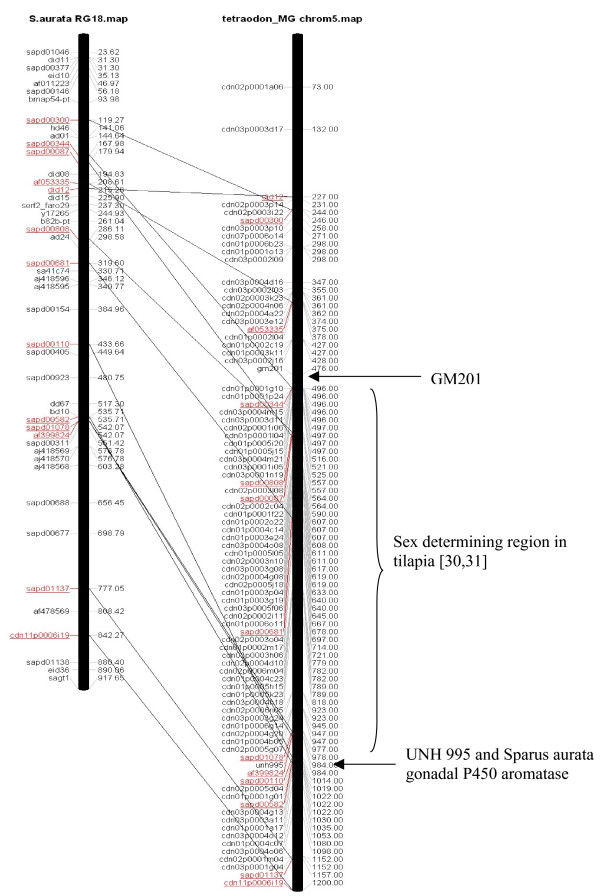
Comparison of *Sparus aurata *radiation hybrid group 18 with *Tetraodon *chromosome 5 and virtual mapping of sea bream ESTs on *Tetraodon *chromosome 5.

Mapping more EST sequences on the RH map confirmed the well-conserved synteny between gilthead sea bream *Sparus aurata *and the pufferfish *Tetraodon nigroviridis*. Recently a large number of new ESTs sequences were obtained from several different cDNA libraries by the Marine Genomics Europe project and more sequences are expected from other ongoing European projects, such as AQUAFIRST and WEALTH. In silico mapping of those sequences to the genome of *Tetraodon *(Table 2, Figure 6) can provide a first approximation as to where those transcripts are located in sea bream based on the high conservation of synteny between *Tetraodon *and sea bream genomes. This makes mapping of candidate genes more straightforward and also facilitates the search for conserved functional genome regions.

In order to retrieve information by comparative mapping two approaches were pursued which are described in more detail below. The first approach looked at the molecular markers mapped in sea bream to localize potential candidate genes in the *Tetraodon *genome. In the second approach candidate genes or ESTs available in sea bream were mapped on the *Tetraodon *genome (Table 2) to facilitate primer design in specific candidate regions for growth, disease resistance or sex determination and also to use them in further studies which aimed to result in higher resolution mapping of these radiation hybrid groups.

The standard approach to find a gene in classical genetics is to specify a gene product and then to try to identify the gene. In the field of molecular genetics the reverse approach is applied; genes are identified purely on the basis of their position in the genome through so-called reverse genetics or positional cloning. In the present study in silico RH mapping is demonstrated to identify candidate genes, first by localizing specific functional groups of interest in *Tetraodon *chromosomes, and subsequently to identify the corresponding RH groups in sea bream and to corroborate the findings by *in vitro *RH mapping. Three examples, namely *DMRT1*, gonadal P450 aromatase and cytochrome P450 aromatase are described below for which first in silico positioning was performed and then confirmed by RH mapping with primers designed within the exons of those genes. *DMRT1 *belongs to the highly conserved group of genes containing the DM domain, which may be involved in sex determination [24]. In Teleostei although at least six genes containing the DM domain are found their function is still unknown [25]. Looking at those genes we found that they are localized in chromosome 12 and 1 of *Tetraodon *and chromosome 5 in zebrafish; both *Tetraodon *chromosome 12 and *Danio *chromosome 5 correspond to RH group 16, suggesting that this RH group could be of interest for mapping of QTLs related to sex determination.

The second and third example for in silico mapping is positioned in the sex-determining region of *Tilapia *that was mapped to linkage group 1 in *Tilapia *[26,27]. Linkage group 1 of *Tilapia *corresponds to *Tetraodon *chromosome 5 and *Sparus *RH group 18 (Figure 6). The gene order between *Sparus *RH group 18 and *Tetraodon *chromosome 5 is particularly well conserved compared to the other RH groups and their corresponding *Tetraodon *chromosomes, suggesting another specific region for QTL mapping. In this particularly well conserved region of *Tetraodon *chromosome 5 we found the gene for gonadal P450 aromatase, a neural marker of estrogen effect known to be involved in sex differentiation [28,29] as well as cytochrome P450 aromatase, which catalyzes the key step in estrogen biosynthesis [30,31] and is a neural marker of estrogen effect in teleosts.

The *in vitro *mapping of DM domain genes (*DMRT 1 and 2*), gonadal P450 aromatase and cytochrome P450 aromatase to *Tetraodon *assigned the DM domain genes to *Tetraodon *chromosome 12 and the two P450 aromatases genes to *Tetraodon *chromosome 5. Chromosome 12 and chromosome 5 are the homologues to RH group 16 and RH 18 respectively. In silico mapping corroborated these findings allocating the DM domain genes to RH group 16 and the two P450 aromatases genes to RH group 18. In this way the correspondence between *Sparus aurata *and *Tetraodon *can facilitate the identification of genes corresponding to QTLs.

Finally, by mapping gene-based markers, potential functional units were identified mapping in radiation hybrid groups 16 and 24: on RH16 the *Sparus aurata *prolactin receptor [32], growth hormone receptor [33] and the homologue of osteoclast-stimulating factor and on RH 24 the *Sparus aurata *growth hormone gene [34], prolactin (PRL) [35] and *osteocalcin *gene [36], all of which are candidate genes for growth-related QTLs of potential economic interest.

## Conclusion

**Figure 7:**
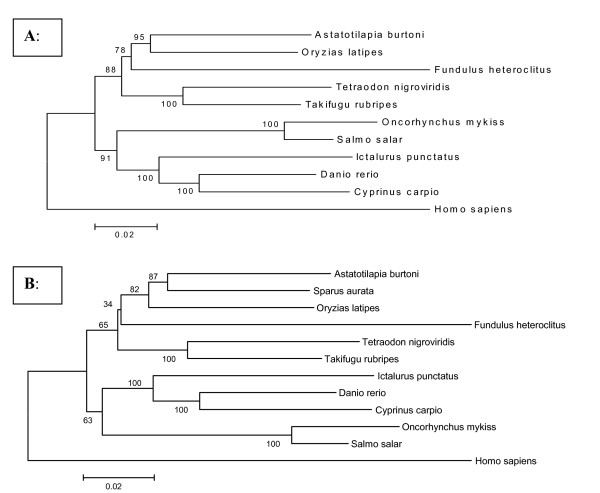
Phylogenetic tree based on a combined dataset of 22 genes modified after [43]. Maximum parsimony (MP) analyses of the combined amino acid alignement were performed with MEGA version 2.1 [44]. Values above branches indicate bootstrap values calculated from maximum parsimony. **A**: Phylogenetic tree based on amino acid alignement from [43] combining 22 genes of ten fish species and human as outgroup B: Phylogenetic tree including the 22 genes of sea bream in the amino acid alignement showing the relationship of seabream to other important model and aquaculture fish species.

By establishing syntentic relationships between *Tetraodon nigroviridis *and *Sparus aurata *through RH mapping of genes combined with all molecular information available today, identification of candidate genes for QTLs in sea bream is more straightforward than it has ever been. More information is expected to come from Medaka (*Oryzias latipes*), for which full sequences information will soon be available, as it appears to be more closely related to sea bream than *Tetraodon nigroviridis *(Figure 7). Furthermore, conserved synteny provides an opportunity for electronically mapping of ESTs to the sea bream RH map first by mapping them to the *Tetraodon *genome. This shortcut will accelerate studies in genome evolution and will give first hints into the genetic make-up of the gilthead sea bream, a species not only of great economical importance but also of considerable evolutionary interest.

## Methods

### RH panel

The RH panel used in the present study has been previously described [18]. Amplification of the RH panel was perfomed four times in parallel using the GenomiPhi Kit (Amersham-Biosciences). Prior to pooling the four amplification reactions each panel was tested with two primer pairs in order to verify the absence of contamination.

### Development of markers

Oligonucleotide primers were designed from sea bream cDNA sequences generated out of five cDNA libraries: mixed embryonic and early larvae library, liver library [37], kidney [32], pituitary [35], 20–135 days post hatch larvae [38], using Primer 3 software [45]. When seabream cDNA aligned to the Genome of *Tetraodon *nigroviridis primer were designed within exons using the Spidey software [39].

### Genotyping

PCR reactions were set up by a Biomek 2000 robot (Beckman) in 96 well microtiter plates. Each PCR reaction had a final volume of 10 μl containing 0.4 μl of forward and reverse primer (20 μM), 1 μl of 10 × PCR buffer (10× PCR buffer contained 100 mM Tris-HCl (pH 8.3), 500 mM KCl, 15 mM MgCl_2 _and 0.1% (w/v) gelatin), 0.02 μl each of 100 mM dATP, dCTP, dGTP and dTTP, 5.82 μl water, 0.1 μl Taq polymerase (5 U/μl, GenAxon) and 2.5 μl of radiation hybrid DNA (approx. 50 ng/μl). After the first denaturation step of 8 min at 94°C, PCR was performed for 20 cycles: 30 s at 94°C, 30 s at the appropriate annealing temperature × (-0,5°C/cycle) for a given primer set and 30 s at 73°C, following those 20 cycles final 15 cycles were performed: 30 s at 94°C, 30 s at × – 10°C and 72 for 30°C. The concluding elongation step was for 5 min at 73°C. The PCR reactions were performed using MWG PCR machines.

### Gel electrophoresis and analysis

PCR products were separated on 2% TBE agarose (Qualex Gold) gels containing 0.01% (v/v) of ethidium bromide solution (10 mg/ml). The gels were poured into gel trays containing 16 combs with 30 wells. The gel run was performed at 200 V (5 V/cm) for 25 min. The gel images were captured by NIH Image 1.61 [46] on a Power Macintosh 8500/120. The macros for gel capturing and semi-automatic analysis was developed by R. Geisler [40].

### Construction of the radiation hybrid map

Bands were scored manually as present (1), absent (0) or unclear (2). In total 960 molecular markers were genotyped. We rejected those markers with no PCR product, or where sea bream and hamster band were not clearly distinguishable. The radiation hybrid analysis was performed for 1,171 molecular markers in total including previously published vectors of [18] using the TSP approach implemented in the rh_tsp_map2 software package in conjunction with the CONCORDE package [41]. Radiation hybrid groups were generated by calculating the pairlods with retention set to the arithmetic mean of pair and all, with an initial LOD score of 3 which was then raised to 6. The resulting data were subsequently analysed by single-linkage clustering in order to obtain radiation groups [41].

### Comparative genomics

BLAT searching was performed using -q = dnax and -t= dnax with a score above 80 and an alignment length of more than 50 bp as recommended for mapping ESTs to the genome across species [42]. Sequences submitted to BLAT searching came from the 937 radiation hybrid mapped ESTs and microsatellites produced within the European project BRIDGE-MAP, (present study and [18]) in addition to 31,705 EST sequences generated by the Marine Genomics Europe network and sequences of selected genes such as genes with a putative role in sex determination downloaded from the NCBI database. BLAST searches were performed using a significance threshold of an alignment length of >50 bp and an e-value of <10^-4 ^(Additional file 3).

## Authors' contributions

**ES **performed main EST production, carried out the genotyping on the RH map, performed map calculation and comparative analysis and conceived design and manuscript writing. **RF and BL **contributed to EST production, alignment and primer design for the molecular markers. **DMP **contributed substantially to the conception and the design of the project and also to manuscript writing. **LB **contributed to the conception and design of the project and to the manuscript writing. **AM **contributed to the conception and design of the project and helped to draft the manuscript. **FS **produced the RH panel. **MT **assisted to develop 20 microsatellite markers. **TP **contributed to the conception and design of the project. **FG **produced the RH panel and contributed to the manuscript writing. **GK: **conceived the study, contributed to the conception and design of the project and also to the manuscript writing. **RG **contributed to the design of the project and to manuscript writing.

All authors read and approved the final manuscript.

## Supplementary Material

Additional File 1 *Appendix 1. Datasheet containing Sparus RH vectors of genotyped markers*. Click here for file

Additional File 2 *Appendix 2*. Datasheet containing markers mapped in the first generation of RH map (sequences coming from NCBI) [18] and new designed primers based on ESTs coming from cDNA libraries produced within the Bridgemap project. Click here for file

Additional File 3 *Appendix3. In silico derived comparative mapping information through BLAT and BLAST *search. Click here for file

## Figures and Tables

**Table 1:** Number of BLAST matches (against ENSEMBL databases [v.38 – Apr2006]) and BLAT matches (against datasets from Genoscope [*Tetraodon nigroviridis *V7, February 2004] and the Wellcome Trust Sanger Institute [*Danio rerio *Zv6, March 2006] including un_random scaffolds) of 794 sea bream sequences mapped on the RH map.

Matches with	BLAST search e<10^-4^	BLAT search score>80
*Tetraodon*	344 (43%)	301 (38%)
*Danio*	243 (30%)	90 (11%)

**Table 2:** BLAT search of 31,705 EST sequences generated by Marine Genomics. BLAT searching was performed using -q = dnax and -t = dnax as recommended for mapping ESTs to the genome across species.

***Tetraodon***	**MG**
**Chr1**	391
**Chr2**	398
**Chr3**	234
**Chr4**	147
**Chr5**	223
**Chr6**	103
**Chr7**	258
**Chr8**	215
**Chr9**	288
**Chr10**	280
**Chr11**	327
**Chr12**	309
**Chr13**	256
**Chr14**	224
**Chr15**	247
**Chr16**	156
**Chr17**	202
**Chr18**	198
**Chr19**	93
**Chr20**	34
**Chr21**	178
**subtotal**	4761
**un_random**	2148
**Total**	6806

Abbreviations: Chr: chromosome; MG: Marine Genomics sequences
