# Supplementary material for: A gene-based radiation hybrid map of the gilthead sea bream Sparus aurata refines and exploits conserved synteny with Tetraodon nigroviridis
Source: BMC Genomics. 2007 Feb 7;8:44. doi: 10.1186/1471-2164-8-44 (PMC1805437; doi:10.1186/1471-2164-8-44)
Supplement: Additional File 2 — Appendix 2. Datasheet containing markers mapped in the first generation of RH map (sequences coming from NCBI) [18] and new designed primers based on ESTs coming from cDNA libraries produced within the Bridgemap project. [file 1471-2164-8-44-S2.doc]

**Appendix 2:** Set of molecular markers for which primers were designed independently for quality testing
