# Supplementary material for: A gene-based radiation hybrid map of the gilthead sea bream Sparus aurata refines and exploits conserved synteny with Tetraodon nigroviridis
Source: BMC Genomics. 2007 Feb 7;8:44. doi: 10.1186/1471-2164-8-44 (PMC1805437; doi:10.1186/1471-2164-8-44)
Supplement: Additional File 3 — Appendix3. In silico derived comparative mapping information through BLAT and BLAST search. [file 1471-2164-8-44-S3.doc]

**BLAST SEARCH against** ENSEMBL database (v.38 - Apr2006)

| **Name** | **Tetraodon chromosome** | **% Identity** | **Alignement length** | **e-value** |
| --- | --- | --- | --- | --- |
| 122_EPPT | 11 | 91.19 | 159 | 3E-38 |
| 128_EPPT | 9 | 83.78 | 74 | 0.00003 |
| 132_EPPT | 3 | 92.48 | 226 | 5E-84 |
| 136_EPPT | 9 | 92.73 | 165 | 2E-59 |
| 1802_Br9D12 | 1 | 90.36 | 83 | 3E-20 |
| 188_EPPT | 6 | 87.68 | 138 | 2E-27 |
| 22_EPPT | 11 | 82.5 | 120 | 3E-11 |
| 220_EPPT | 7 | 96.63 | 208 | 1E-90 |
| 256_EPPT | 2 | 89.47 | 76 | 6E-16 |
| 266_EPPT | 16 | 79.68 | 251 | 2E-16 |
| 275_EPPT | 17 | 85.07 | 67 | 0.000006 |
| 278_EPPT | 4 | 83.05 | 118 | 2E-12 |
| 28_EPPT | 14 | 93.81 | 113 | 2E-40 |
| 293_EPPT | 8 | 90.91 | 77 | 4E-17 |
| 306_KD7G01 | 2 | 86.36 | 154 | 2E-31 |
| 317_Sub23 | 17 | 87.94 | 141 | 3E-33 |
| 414_A11 | 13 | 89.89 | 89 | 4E-19 |
| 59_EPPT | 12 | 89.66 | 58 | 2E-08 |
| 606_ICPT | 2 | 91.3 | 299 | 1E-103 |
| 614_ICPT | 16 | 90.32 | 93 | 2E-21 |
| 620_ICPT | 15 | 88.76 | 258 | 2E-74 |
| 67_EPPT | 18 | 80.49 | 205 | 1E-16 |
| 712_PIL1B9 | 2 | 88.24 | 102 | 6E-22 |
| 980_KD7D05 | 18 | 86.81 | 182 | 6E-41 |
| AF013105 | 3 | 92.14 | 229 | 7E-84 |
| AF013120 | 16 | 91.07 | 56 | 5E-11 |
| AF047467 | 2 | 87.31 | 134 | 2E-29 |
| AF083556 | 2 | 87.88 | 99 | 4E-20 |
| AF136979 | 14 | 88.11 | 185 | 7E-47 |
| AF136980 | 14 | 87.64 | 178 | 7E-43 |
| AF149756 | 3 | 84.65 | 202 | 2E-22 |
| AF150904 | 2 | 94.62 | 186 | 2E-76 |
| AF151718 | 2 | 84.58 | 240 | 3E-44 |
| AF169368 | 12 | 82.08 | 212 | 5E-25 |
| AF184170 | 21 | 90.13 | 304 | 4E-99 |
| AF197904 | 13 | 82.07 | 184 | 3E-20 |
| AF210428 | 1 | 88.33 | 60 | 4E-09 |
| AF253527 | 12 | 81.58 | 114 | 5E-07 |
| AF258447 | 3 | 83.39 | 313 | 1E-51 |
| AF300425 | 10 | 88.49 | 139 | 4E-32 |
| AF316853 | 14 | 93.55 | 124 | 2E-40 |
| AF377998 | 2 | 83.55 | 152 | 1E-20 |
| AF399824 | 5 | 84.64 | 267 | 2E-49 |
| AF438176 | 12 | 86.11 | 180 | 9E-37 |
| AF478568 | 19 | 82.05 | 234 | 5E-26 |
| AF478569 | 19 | 83.75 | 80 | 0.00001 |
| AF491302 | 11 | 89.95 | 189 | 1E-56 |
| AF510084 | 16 | 87.82 | 156 | 1E-36 |
| AF520608 | 3 | 82.51 | 223 | 3E-29 |
| AJ276406 | 11 | 84.18 | 158 | 2E-24 |
| AJ289717 | 7 | 85.54 | 1134 | 0 |
| AJ291667 | 16 | 89.94 | 159 | 5E-41 |
| AJ418543 | 9 | 83.78 | 148 | 1E-20 |
| AJ418544 | 3 | 86.96 | 161 | 1E-35 |
| AJ418571 | 14 | 92.54 | 67 | 8E-18 |
| AJ418572 | 14 | 91.23 | 57 | 6E-12 |
| AJ418573 | 10 | 89.47 | 76 | 1E-16 |
| AJ418580 | 2 | 86.67 | 105 | 4E-19 |
| AJ418584 | 1 | 82.99 | 194 | 5E-22 |
| AJ418585 | 1 | 86.96 | 92 | 6E-16 |
| AJ418593 | 12 | 81.19 | 101 | 0.0001 |
| AJ418595 | 5 | 86.96 | 69 | 2E-09 |
| AJ418605 | 12 | 83.16 | 95 | 0.00001 |
| AJ418616 | 7 | 91.45 | 117 | 1E-35 |
| AJ418622 | 1 | 84.06 | 69 | 0.00001 |
| AJ418626 | 12 | 94.83 | 58 | 6E-17 |
| AJ418633 | 21 | 84.52 | 84 | 7E-09 |
| AJ418654 | 13 | 93.38 | 151 | 2E-49 |
| AJ418660 | 14 | 94.92 | 197 | 9E-83 |
| AJ418662 | 2 | 92.86 | 70 | 1E-19 |
| AJ418663 | 7 | 95.83 | 96 | 2E-37 |
| AJ418668 | 14 | 98.1 | 105 | 1E-47 |
| AJ418672 | 9 | 84.62 | 78 | 1E-07 |
| AJ418673 | 18 | 82.88 | 146 | 9E-15 |
| AJ418680 | 17 | 84.07 | 113 | 5E-14 |
| AJ418690 | 21 | 89.09 | 55 | 1E-08 |
| AJ418691 | 20 | 91.11 | 90 | 3E-24 |
| AJ418693 | 10 | 95.3 | 149 | 5E-62 |
| AJ418695 | 8 | 88.96 | 163 | 2E-40 |
| AJ418698 | 18 | 88.34 | 163 | 7E-42 |
| AJ419178 | 12 | 80.99 | 142 | 2E-09 |
| AY046314 | 3 | 90.05 | 382 | 1E-126 |
| AY173039 | 15 | 91.43 | 140 | 1E-37 |
| AY246695 | 2 | 85.48 | 186 | 1E-35 |
| AY326430 | 3 | 90.12 | 881 | 0 |
| Bd36 | 21 | 95.18 | 228 | 9E-101 |
| Bd72 | 2 | 90.91 | 99 | 5E-25 |
| Bmap42PT | 21 | 90.18 | 112 | 2E-30 |
| Bmap4PT | 11 | 86.15 | 65 | 2E-07 |
| Bt39PT | 4 | 90.1 | 101 | 3E-26 |
| cDN01P0001I11 | 3 | 89.78 | 137 | 3E-38 |
| cDN01P0002K06 | 11 | 91.26 | 103 | 1E-29 |
| cDN01P0004P04 | 14 | 84.88 | 86 | 7E-10 |
| cDN02P0001E11 | 16 | 95.24 | 441 | 0 |
| cDN02P0001J08 | 7 | 84 | 125 | 3E-15 |
| cDN02P0002I18 | 2 | 89.79 | 235 | 1E-62 |
| cDN02P0004A14 | 17 | 85.07 | 67 | 0.000008 |
| cDN02P0004D09 | 13 | 85.86 | 198 | 6E-41 |
| cDN02P0005H21 | 7 | 86.11 | 108 | 3E-18 |
| cDN02P0005L19 | 12 | 88.54 | 96 | 9E-21 |
| cDN02P0006M09 | 7 | 85.95 | 121 | 2E-21 |
| cDN03P0002G13 | 10 | 90.43 | 188 | 6E-59 |
| cDN03P0004L05 | 19 | 90.57 | 159 | 1E-48 |
| cDN03P0005E06 | 6 | 86.9 | 145 | 8E-31 |
| cDN03P0006F11 | 13 | 88.79 | 116 | 5E-26 |
| cDN04P0001G13 | 2 | 85.82 | 134 | 3E-24 |
| cDN04P0003J01 | 11 | 87.37 | 95 | 8E-18 |
| cDN04P0006M10 | 1 | 91.71 | 422 | 7E-158 |
| cDN05P0001F01 | 9 | 90.77 | 65 | 3E-14 |
| cDN05P0001L04 | 15 | 87.29 | 118 | 2E-24 |
| cDN05P0001O09 | 10 | 82.83 | 99 | 2E-08 |
| cDN05P0003M07 | 2 | 87.5 | 200 | 2E-49 |
| cDN05P0004N23 | 15 | 86.07 | 122 | 7E-22 |
| cDN05P0005J07 | 1 | 89.23 | 130 | 2E-31 |
| cDN05P0006A01 | 1 | 88.11 | 143 | 1E-34 |
| cDN06_CL213 | 1 | 91.48 | 458 | 2E-164 |
| cDN06P0004F01 | 15 | 89.94 | 159 | 2E-46 |
| cDN06P0006I09 | 11 | 87.06 | 85 | 4E-14 |
| cDN07P0004A03 | 7 | 91.11 | 180 | 7E-59 |
| cDN07P0006O14 | 5 | 93.18 | 88 | 6E-28 |
| cDN08P0006E16 | 3 | 90.09 | 111 | 2E-27 |
| cDN09P0005N14 | 11 | 91.22 | 148 | 6E-47 |
| cDN10P0001M15 | 10 | 83.98 | 437 | 5E-77 |
| cDN10P0003H19 | 3 | 84.07 | 113 | 4E-14 |
| cDN11_CL163 | 14 | 82.91 | 117 | 1E-11 |
| cDN11_CL42 | 13 | 84.85 | 99 | 2E-13 |
| cDN11P0001I12 | 9 | 91.49 | 94 | 9E-27 |
| cDN11P0003J02 | 3 | 85.64 | 188 | 2E-37 |
| cDN11P0003L16 | 16 | 85.33 | 75 | 2E-08 |
| cDN11P0004L03 | 6 | 89.83 | 236 | 3E-73 |
| cDN11P0005C06 | 17 | 85.86 | 99 | 3E-15 |
| cDN11P0006I19 | 5 | 86.92 | 130 | 1E-26 |
| cDN11P0006K15 | 2 | 82.82 | 163 | 5E-20 |
| cDN12P0006G10 | 13 | 90.98 | 122 | 4E-36 |
| cDN12P0006J21 | 12 | 83.16 | 95 | 4E-08 |
| cDN13P0001J14 | 10 | 88.1 | 84 | 6E-16 |
| cDN13P0004P17 | 21 | 85.61 | 139 | 6E-25 |
| cDN13P0005C21 | 6 | 92.16 | 102 | 1E-31 |
| cDN14_CL101 | 15 | 89.29 | 56 | 7E-09 |
| cDN14_CL77 | 11 | 83.54 | 158 | 1E-17 |
| cDN14P0001A18 | 11 | 85.32 | 109 | 2E-16 |
| cDN14P0002G12 | 8 | 92.41 | 79 | 1E-22 |
| cDN14P0005N21 | 12 | 89.73 | 146 | 3E-41 |
| cDN14P0006E20 | 3 | 93.41 | 91 | 1E-29 |
| cDN14P0006P04 | 8 | 87.29 | 291 | 1E-69 |
| Cld35 | 1 | 81.31 | 107 | 0.0002 |
| Cld52 | 13 | 93.04 | 158 | 7E-58 |
| Cld84 | 13 | 85.16 | 128 | 9E-21 |
| Dd77 | 6 | 94.63 | 149 | 2E-60 |
| Dd84 | 18 | 90 | 80 | 3E-16 |
| Dd91 | 14 | 85.71 | 140 | 3E-23 |
| DId12 | 5 | 93.6 | 125 | 2E-45 |
| DId14 | 1 | 88.78 | 98 | 3E-22 |
| Dt93PT | 19 | 87.88 | 99 | 3E-16 |
| Ed74PT | 7 | 91.38 | 58 | 1E-12 |
| Ed84PT | 8 | 86.13 | 310 | 1E-68 |
| Ed93PT | 4 | 89.06 | 64 | 6E-12 |
| Et10PT | 14 | 91.94 | 124 | 1E-37 |
| Et5PT | 11 | 87.04 | 54 | 0.00001 |
| Fd46 | 12 | 91.47 | 129 | 1E-40 |
| Fd48 | 12 | 95.16 | 62 | 1E-19 |
| G4 | 2 | 92.86 | 70 | 1E-19 |
| G6 | 7 | 95.83 | 96 | 2E-37 |
| G6tetra | 7 | 95.83 | 96 | 2E-37 |
| Gd67 | 6 | 88.89 | 99 | 2E-22 |
| Gt57PT | 7 | 96.1 | 77 | 1E-28 |
| Hd15 | 3 | 97.42 | 194 | 9E-94 |
| Hd28 | 1 | 86.59 | 82 | 1E-12 |
| SAPD00003 | 11 | 85.23 | 149 | 3E-26 |
| SAPD00007 | 3 | 83.33 | 168 | 3E-23 |
| SAPD00015 | 13 | 87.76 | 98 | 1E-19 |
| SAPD00024 | 9 | 87.72 | 171 | 1E-41 |
| SAPD00031 | 17 | 86.86 | 137 | 1E-28 |
| SAPD00035 | 13 | 90 | 140 | 5E-40 |
| SAPD00039 | 2 | 90.48 | 231 | 9E-73 |
| SAPD00043 | 11 | 92.59 | 54 | 5E-10 |
| SAPD00060 | 2 | 85.83 | 120 | 7E-21 |
| SAPD00062 | 1 | 90.11 | 364 | 2E-95 |
| SAPD00066 | 15 | 93.69 | 111 | 1E-37 |
| SAPD00080 | 15 | 84.46 | 148 | 3E-23 |
| SAPD00087 | 5 | 88.68 | 53 | 4E-07 |
| SAPD00091 | 11 | 88.18 | 220 | 4E-59 |
| SAPD00094 | 3 | 87.72 | 171 | 1E-41 |
| SAPD00110 | 5 | 89.33 | 178 | 6E-38 |
| SAPD00112 | 19 | 90.73 | 248 | 2E-64 |
| SAPD00116 | 11 | 85.61 | 132 | 3E-23 |
| SAPD00117 | 18 | 85.25 | 61 | 0.00009 |
| SAPD00118 | 2 | 89.12 | 147 | 2E-39 |
| SAPD00133 | 19 | 90.83 | 120 | 6E-35 |
| SAPD00136 | 14 | 82.84 | 134 | 6E-15 |
| SAPD00150 | 15 | 88.68 | 53 | 4E-07 |
| SAPD00154 | 5 | 90.62 | 224 | 2E-73 |
| SAPD00158 | 3 | 82.58 | 155 | 6E-18 |
| SAPD00159 | 9 | 87.67 | 146 | 5E-34 |
| SAPD00165 | 13 | 88.16 | 76 | 1E-13 |
| SAPD00172 | 3 | 90.37 | 135 | 2E-39 |
| SAPD00174 | 2 | 91.27 | 126 | 8E-39 |
| SAPD00175 | 19 | 94.74 | 152 | 2E-61 |
| SAPD00185 | 9 | 82.63 | 167 | 3E-20 |
| SAPD00210 | 17 | 84.11 | 107 | 4E-13 |
| SAPD00221 | 15 | 94.35 | 177 | 7E-72 |
| SAPD00232 | 18 | 94.78 | 134 | 1E-50 |
| SAPD00242 | 6 | 92.86 | 154 | 4E-53 |
| SAPD00254 | 9 | 87.5 | 144 | 8E-33 |
| SAPD00278 | 9 | 85.98 | 107 | 7E-18 |
| SAPD00300 | 5 | 92.21 | 154 | 4E-53 |
| SAPD00301 | 7 | 89.13 | 138 | 2E-36 |
| SAPD00302 | 21 | 87.21 | 86 | 7E-15 |
| SAPD00308 | 11 | 85.91 | 220 | 4E-47 |
| SAPD00311 | 13 | 84.07 | 113 | 3E-14 |
| SAPD00317 | 17 | 87.8 | 123 | 2E-27 |
| SAPD00324 | 12 | 83.12 | 160 | 7E-21 |
| SAPD00332 | 9 | 85.85 | 205 | 6E-43 |
| SAPD00344 | 5 | 86.17 | 188 | 6E-40 |
| SAPD00349 | 11 | 84.31 | 102 | 2E-12 |
| SAPD00352 | 10 | 85.51 | 69 | 4E-07 |
| SAPD00356 | 1 | 92.92 | 113 | 4E-38 |
| SAPD00358 | 15 | 85.54 | 83 | 4E-07 |
| SAPD00364 | 18 | 82.07 | 145 | 3E-14 |
| SAPD00366 | 1 | 88.75 | 160 | 2E-42 |
| SAPD00370 | 17 | 83.81 | 488 | 2E-92 |
| SAPD00372 | 15 | 84.62 | 78 | 1E-07 |
| SAPD00378 | 1 | 83.17 | 208 | 9E-33 |
| SAPD00385 | 10 | 90.67 | 75 | 4E-13 |
| SAPD00394 | 16 | 86.34 | 161 | 2E-33 |
| SAPD00401 | 3 | 85.71 | 63 | 0.000006 |
| SAPD00403 | 12 | 90.91 | 55 | 2E-10 |
| SAPD00405 | 14 | 84.88 | 86 | 4E-10 |
| SAPD00413 | 9 | 80.56 | 144 | 6E-09 |
| SAPD00415 | 10 | 83.89 | 211 | 3E-32 |
| SAPD00427 | 6 | 87.8 | 123 | 2E-27 |
| SAPD00447 | 17 | 91.76 | 85 | 2E-21 |
| SAPD00453 | 8 | 88.41 | 164 | 4E-42 |
| SAPD00462 | 11 | 88.29 | 111 | 5E-25 |
| SAPD00466 | 10 and 2 | 84.44 | 135 | 5E-19 |
| SAPD00470 | 1 | 88.95 | 190 | 8E-53 |
| SAPD00478 | 2 | 87.58 | 153 | 9E-36 |
| SAPD00488 | 18 | 83.65 | 104 | 3E-11 |
| SAPD00494 | 2 | 90.18 | 163 | 7E-49 |
| SAPD00496 | 13 | 81.2 | 133 | 2E-09 |
| SAPD00498 | 1 | 86.96 | 115 | 1E-22 |
| SAPD00505 | 11 | 92.56 | 121 | 1E-40 |
| SAPD00510 | 6 | 92.45 | 53 | 5E-12 |
| SAPD00519 | 13 | 94.5 | 109 | 2E-38 |
| SAPD00523 | 3 | 85.44 | 158 | 3E-29 |
| SAPD00526 | 2 | 91.12 | 169 | 7E-55 |
| SAPD00531 | 18 | 89.88 | 168 | 1E-49 |
| SAPD00548 | 12 | 84.09 | 220 | 1E-37 |
| SAPD00553 | 13 | 87.43 | 167 | 2E-39 |
| SAPD00556 | 2 | 83 | 100 | 0.0004 |
| SAPD00558 | 15 | 86.13 | 137 | 3E-26 |
| SAPD00560 | 9 | 83.33 | 96 | 6E-09 |
| SAPD00562 | 10 | 86.15 | 65 | 4E-07 |
| SAPD00563 | 18 | 86.49 | 148 | 2E-30 |
| SAPD00573 | 21 | 93 | 200 | 7E-76 |
| SAPD00578 | 4 | 87.18 | 117 | 1E-23 |
| SAPD00579 | 1 | 89.17 | 120 | 2E-30 |
| SAPD00581 | 4 | 81.77 | 192 | 7E-21 |
| SAPD00582 | 5 | 90.06 | 161 | 4E-45 |
| SAPD00605 | 1 | 89.51 | 143 | 2E-39 |
| SAPD00619 | 12 | 85.87 | 92 | 1E-13 |
| SAPD00631 | 18 | 90.7 | 129 | 4E-38 |
| SAPD00637 | 12 | 89.74 | 78 | 3E-17 |
| SAPD00638 | 14 | 88.04 | 184 | 4E-47 |
| SAPD00640 | 3 | 93.59 | 78 | 2E-22 |
| SAPD00642 | 3 | 92.97 | 128 | 1E-44 |
| SAPD00647 | 18 | 88.89 | 135 | 1E-34 |
| SAPD00651 | 7 | 85.71 | 56 | 0.0004 |
| SAPD00660 | 13 | 83.33 | 168 | 3E-23 |
| SAPD00665 | 2 | 92.07 | 164 | 1E-56 |
| SAPD00676 | 8 | 84.04 | 213 | 1E-35 |
| SAPD00677 | 5 | 90.59 | 85 | 2E-21 |
| SAPD00681 | 5 | 86.36 | 88 | 1E-13 |
| SAPD00688 | 13 | 83.33 | 72 | 0.0004 |
| SAPD00702 | 15 | 88.29 | 222 | 2E-58 |
| SAPD00707 | 1 | 88.46 | 52 | 0.000001 |
| SAPD00714 | 11 | 83.33 | 186 | 7E-27 |
| SAPD00715 | 8 | 91.54 | 130 | 3E-41 |
| SAPD00717 | 9 | 86.92 | 130 | 7E-27 |
| SAPD00726 | 8 | 90.3 | 134 | 9E-39 |
| SAPD00735 | 12 | 86.13 | 137 | 3E-26 |
| SAPD00767 | 15 | 82.52 | 103 | 3E-08 |
| SAPD00774 | 9 | 88.61 | 158 | 4E-41 |
| SAPD00798 | 14 | 89.62 | 106 | 3E-27 |
| SAPD00802 | 4 | 86.11 | 108 | 2E-18 |
| SAPD00808 | 5 | 86.57 | 67 | 0.000006 |
| SAPD00816 | 12 | 86.59 | 164 | 3E-35 |
| SAPD00832 | 14 | 90.98 | 122 | 3E-36 |
| SAPD00833 | 8 | 86.67 | 135 | 7E-28 |
| SAPD00835 | 13 | 82.76 | 116 | 3E-11 |
| SAPD00863 | 2 | 86.08 | 158 | 2E-31 |
| SAPD00865 | 9 | 81.07 | 317 | 1E-35 |
| SAPD00868 | 13 | 87.5 | 160 | 2E-37 |
| SAPD00877 | 7 | 84.93 | 146 | 3E-24 |
| SAPD00889 | 16 | 90.28 | 144 | 8E-43 |
| SAPD00890 | 12 | 91.3 | 184 | 2E-61 |
| SAPD00903 | 14 | 85.33 | 75 | 3E-08 |
| SAPD00905 | 9 | 85.58 | 104 | 9E-16 |
| SAPD00919 | 16 | 82.22 | 90 | 0.00004 |
| SAPD00929 | 13 | 88.89 | 54 | 1E-07 |
| SAPD00935 | 18 | 93.78 | 209 | 2E-83 |
| SAPD00947 | 21 | 87.04 | 54 | 0.00004 |
| SAPD00950 | 8 | 88.46 | 234 | 4E-65 |
| SAPD00956 | 7 | 88.56 | 201 | 1E-54 |
| SAPD00958 | 1 | 89.75 | 244 | 2E-75 |
| SAPD00960 | 11 | 86.25 | 349 | 1E-78 |
| SAPD00964 | 2 | 89.33 | 225 | 1E-63 |
| SAPD00965 | 13 | 89.14 | 175 | 8E-49 |
| SAPD00970 | 3 | 92.97 | 128 | 1E-44 |
| SAPD00972 | 7 | 90.13 | 152 | 1E-44 |
| SAPD00978 | 15 | 90 | 50 | 1E-07 |
| SAPD00979 | 12 | 84.4 | 109 | 3E-14 |
| SAPD00990 | 12 | 89.43 | 123 | 4E-32 |
| SAPD00993 | 13 | 90.91 | 132 | 6E-40 |
| SAPD01003 | 2 | 88.6 | 114 | 1E-26 |
| SAPD01010 | 9 | 86.72 | 128 | 1E-25 |
| SAPD01020 | 10 | 92.5 | 160 | 1E-56 |
| SAPD01023 | 12 | 88.68 | 53 | 0.0001 |
| SAPD01029 | 9 | 85.35 | 157 | 2E-28 |
| SAPD01054 | 16 | 81.07 | 544 | 2E-68 |
| SAPD01068 | 6 | 85.53 | 159 | 3E-30 |
| SAPD01076 | 19 | 87.78 | 180 | 1E-44 |
| SAPD01077 | 8 | 87.65 | 81 | 4E-14 |
| SAPD01078 | 5 | 89.36 | 94 | 2E-22 |
| SAPD01079 | 7 | 82.88 | 111 | 1E-10 |
| SAPD01087 | 20 | 88.09 | 403 | 7E-108 |
| SAPD01093 | 2 | 84.83 | 178 | 2E-31 |
| SAPD01096 | 20 | 92.81 | 153 | 9E-55 |
| SAPD01097 | 16 | 82.99 | 194 | 5E-27 |
| SAPD01099 | 10 | 92.16 | 102 | 1E-31 |
| SAPD01107 | 9 | 92.59 | 54 | 1E-12 |
| SAPD01108 | 9 | 92.68 | 123 | 2E-41 |
| SAPD01124 | 3 | 90.59 | 170 | 9E-51 |
| SAPD01126 | 15 | 85.56 | 90 | 2E-12 |
| SAPD01128 | 15 | 82.44 | 131 | 3E-13 |
| SAPD01136 | 11 | 85.71 | 70 | 8E-08 |
| SAPD01137 | 5 | 83.66 | 410 | 2E-74 |
| SAPD01139 | 2 | 84.89 | 139 | 1E-22 |
| Sau0009A08 | 2 | 92.42 | 66 | 3E-17 |
| SauG46INRA | 2 | 90.45 | 178 | 9E-51 |
| SpauPK713 | 6 | 87.78 | 90 | 7E-17 |
| SpauPP1C | 6 | 89.83 | 59 | 3E-10 |
| U30311 | 17 | 84.8 | 204 | 2E-33 |
| SAPD00064 | 13 | 94.41 | 143 | 5.00E-56 |

**BLAT SEARCH against** ENSEMBL database (v.38 - Apr2006) (Kent 2002)

| **Name** | **Tetraodon chromosome** | **Score** |
| --- | --- | --- |
| SAPD01079 | 7 | 78 |
| SAPD00069 | 1 | 78 |
| SAPD00210 | 17 | 78 |
| 325_KD7H08 | Un_random | 79 |
| SAPD00401 | 3 | 79 |
| SAPD00324 | 12 | 80 |
| AJ418570 | Un_random | 81 |
| AJ418677 | Un_random | 81 |
| cDN04P0003J01 | Un_random | 81 |
| SAPD00555 | Un_random | 81 |
| SAPD00688 | 13 | 82 |
| SAPD00681 | 5 | 83 |
| SAPD00492 | Un_random | 84 |
| SAPD00808 | 5 | 84 |
| AJ418672 | 9 | 85 |
| Bmap42-PT | 21 | 85 |
| Cld10 | Un_random | 85 |
| Fd79 | Un_random | 85 |
| SAPD00015 | 13 | 85 |
| SAPD00637 | 12 | 86 |
| Ct43-PT | 18 REPEATS | 87 |
| cDN02P0005L19 | 12 | 88 |
| AJ418636 | 10 | 89 |
| SAPD00596 | 7 | 89 |
| Sai19 | Un_random | 90 |
| SAPD01107 | 9 | 90 |
| AJ418571 | 14 | 91 |
| AJ418616 | 7 | 91 |
| DId14 | 1 | 92 |
| EId39 | Un_random | 92 |
| SAPD00466 | 2 | 94 |
| cDN02P0006M09 | 7 | 95 |
| SAPD00767 | 15 | 96 |
| 293_EP-PT | 8 | 97 |
| SAPD00798 | 14 | 97 |
| SAPD00623 | Un_random | 98 |
| cDN14_CL101 | 1 | 100 |
| SAPD00235 | 21_random | 100 |
| 190_EP-PT | Un_random | 101 |
| AJ418622 | 1 | 104 |
| AJ418633 | 21 | 104 |
| SAPD00066 | 15 | 105 |
| SAPD00498 | 1 | 105 |
| SAPD01108 | 9 | 105 |
| AJ418690 | 21 | 106 |
| SAPD01139 | 2 | 106 |
| SAPD00254 | 9 | 107 |
| Dd41 | 15_random | 108 |
| Dd84 | 18 | 108 |
| SAPD00366 | 1 | 108 |
| SAPD00563 | 18 | 108 |
| AJ418662 | 2 | 109 |
| G4 | 2 | 109 |
| SAPD00708 | 11 | 109 |
| AJ418626 | 12 | 111 |
| Cld35 | 1 | 111 |
| SAPD00579 | 1 | 111 |
| Ad21 | 13 | 112 |
| Ad12 | Un_random | 113 |
| SAPD00385 | 10 | 113 |
| SAPD01068 | 6 | 113 |
| SAPD00461 | Un_random | 114 |
| SAPD00415 | 10 | 117 |
| SAPD00640 | 3 | 117 |
| 207_EP-PT | Un_random | 118 |
| 47_EP-PT | Un_random | 119 |
| Et10-PT | 14 | 119 |
| SAPD00364 | 18 | 119 |
| SAPD00043 | 11 | 120 |
| AJ418668 | 14 | 121 |
| cDN05P0001O09 | 10 | 122 |
| SAPD00242 | 6 | 122 |
| SAPD00423 | Un_random | 123 |
| SAPD00660 | 13 | 123 |
| SAPD00003 | 11 | 125 |
| SAPD00308 | 11 | 126 |
| SAPD00903 | 14 | 126 |
| Dd56 | Un_random | 127 |
| AJ418572 | 14 | 128 |
| SAPD00605 | 1 | 128 |
| SAPD00581 | 4 | 129 |
| DId31 | 12 | 130 |
| SAPD01078 | 5 | 130 |
| EId41 | Un_random | 131 |
| C92b-PT | Un_random | 132 |
| AJ419178 | 12 | 134 |
| SAPD00558 | 15 | 134 |
| AJ418663 | 7 | 135 |
| SAPD01136 | 11 | 135 |
| Hd23 | Un_random | 136 |
| Hd28 | 15_random | 136 |
| G6 | 7 | 137 |
| G6tetra | 7 | 137 |
| 59_EP-PT | 12 | 138 |
| AJ418654 | 13 | 138 |
| AJ418698 | 18 | 138 |
| Fd46 | 12 | 138 |
| SAPD00531 | 18 | 138 |
| 1802_Br9D12 | 1 | 139 |
| SAPD00199 | Un_random | 140 |
| cDN05P0003M07 | 2 | 144 |
| SAPD00225 | 12 | 144 |
| Fd48 | 12 | 145 |
| SAPD00833 | 8 | 145 |
| 184_EP-PT | Un_random | 146 |
| SAPD00508 | Un_random | 146 |
| 278_EP-PT | 4 | 147 |
| SAPD00969 | Un_random | 150 |
| 279_EP-PT | Un_random | 151 |
| Dd77 | 6 | 151 |
| SAPD00087 | 5 | 151 |
| AJ418580 | 2 | 154 |
| SAPD00447 | 17 | 155 |
| cDN14_CL77 | 11 | 156 |
| SAPD00587 | 1_random | 156 |
| SAPD00496 | 13 | 157 |
| SAPD00505 | 11 | 157 |
| SAPD00905 | 9 | 158 |
| SAPD01126 | 15 | 158 |
| DId12 | 5 | 159 |
| 266_EP-PT | 16 | 160 |
| SAPD00302 | 21 | 160 |
| SAPD01077 | 8 | 164 |
| SAPD00352 | 10 | 165 |
| Ad66 | Un_random | 166 |
| AF053335 | 5 | 166 |
| SAPD00060 | 2 | 166 |
| AJ418584 | 1 | 167 |
| cDN04P0001G13 | 2 | 168 |
| cDN13P0005C21 | 6 | 171 |
| SAPD00136 | 14 | 171 |
| SAPD00548 | 12 | 171 |
| SAPD00553 | 13 | 173 |
| SAPD01128 | 15 | 177 |
| SAPD00717 | 9 | 178 |
| 614_IC-PT | 16 | 179 |
| cDN01P0002K06 | 11 | 181 |
| SAPD00523 | 3 | 182 |
| Saimbb3 | 1_random | 185 |
| SAPD00118 | 2 | 187 |
| 28_EP-PT | 14 | 188 |
| SAPD00865 | 9 | 188 |
| SAPD00673 | Un_random | 189 |
| AJ418651 | 1_random | 192 |
| Saimbb14 | 1_random | 192 |
| cDN14P0006E20 | 3 | 193 |
| Cld52 | 13 | 193 |
| SAPD01099 | 10 | 194 |
| cDN13P0005D10 | Un_random | 195 |
| SAPD00835 | 13 | 195 |
| AJ276406 | 11 | 196 |
| SAPD00116 | 11 | 196 |
| SAPD01097 | 16 | 197 |
| Bd72 | 2 | 198 |
| Gd46 | 2_random | 198 |
| SAPD00266 | Un_random | 198 |
| SAPD00356 | 1 | 198 |
| AF520608 | 3 | 199 |
| cDN10P0003H19 | 3 | 200 |
| SAPD00868 | 13 | 200 |
| SAPD00344 | 5 | 202 |
| SAPD00631 | 18 | 203 |
| SAPD00462 | 11 | 204 |
| SAPD00519 | 13 | 207 |
| cDN14P0002G12 | 8 | 208 |
| SAPD00714 | 11 | 208 |
| cDN03P0006F11 | 13 | 209 |
| SAPD00494 | 2 | 209 |
| SAPD00006 | 2_random | 210 |
| SAPD00190 | Un_random | 211 |
| cDN05P0006A01 | 1 | 212 |
| SAPD00832 | 14 | 212 |
| SAPD00026 | Un_random | 213 |
| SAPD00642 | 3 | 213 |
| SAPD00031 | 17 | 214 |
| cDN14P0005N21 | 12 | 216 |
| SAPD00573 | 21 | 216 |
| SAPD00582 | 5 | 218 |
| SAPD00647 | 18 | 219 |
| AJ418671 | 1_random | 220 |
| SAPD00569 | 1_random | 220 |
| SAPD00255 | Un_random | 222 |
| SAPD00931 | Un_random | 222 |
| SAPD00405 | Un_random | 224 |
| SAPD01081 | Un_random | 227 |
| 67_EP-PT | 18 | 229 |
| SAPD00611 | Un_random | 229 |
| SAPD01092 | Un_random | 231 |
| cDN06P0006I09 | 11 | 232 |
| Hd15 | 3 | 233 |
| SAPD00453 | 8 | 233 |
| cDN09P0002E08 | Un_random | 234 |
| SAPD00174 | 2 | 234 |
| 22_EP-PT | 11 | 236 |
| cDN11P0006K15 | 2 | 239 |
| SAPD00007 | 3 | 241 |
| AJ418670 | Un_random | 244 |
| SAPD00770 | Un_random | 244 |
| SAPD00950 | 8 | 244 |
| SAPD01137 | 5 | 244 |
| SAPD00702 | 15 | 245 |
| Sau0009A08 | 2 | 245 |
| AJ413189 | Un_random | 246 |
| SAPD00478 | 2 | 247 |
| SAPD00665 | 2 | 250 |
| cDN06P0004F01 | 15 | 251 |
| SAPD00300 | 5 | 251 |
| SAPD00619 | 12 | 251 |
| cDN01P0001I11 | 3 | 252 |
| SAPD00562 | Un_random | 252 |
| 122_EP-PT | 11 | 253 |
| SAPD00911 | Un_random | 255 |
| cDN05P0001L04 | 15 | 256 |
| 127_EP-PT | Un_random | 257 |
| cDN11P0005K16 | Un_random | 258 |
| SAPD00956 | 7 | 258 |
| SAPD00439 | Un_random | 262 |
| SAPD00715 | 8 | 262 |
| SAPD00388 | Un_random | 265 |
| cDN14P0006P04 | 8 | 273 |
| SAPD00529 | 1_random | 275 |
| SAPD01093 | 2 | 275 |
| SAPD00823 | Un_random | 277 |
| cDN12P0006J21 | 12 | 281 |
| SAPD00965 | 13 | 281 |
| 132_EP-PT | 3 | 285 |
| SAPD00317 | 17 | 285 |
| SAPD00860 | Un_random | 286 |
| SAPD00336 | Un_random | 287 |
| SAPD00638 | 14 | 288 |
| cDN13P0004P17 | 21 | 290 |
| Bd36 | 21 | 291 |
| SAPD01112 | Un_random | 291 |
| AY173039 | 15 | 292 |
| cDN02P0005H21 | 7 | 293 |
| SAPD00657 | Un_random | 293 |
| SAPD00774 | 9 | 293 |
| SAPD00232 | 18 | 294 |
| SAPD00035 | 13 | 304 |
| SAPD00972 | 7 | 304 |
| SAPD00877 | 7 | 305 |
| 306_KD7G01 | 2 | 306 |
| SAPD00146 | Un_random | 306 |
| cDN11P0003J02 | 3 | 307 |
| cDN10P0001M15 | 10 | 308 |
| SAPD00159 | 9 | 308 |
| SAPD00039 | 2 | 309 |
| SAPD00112 | 19 | 309 |
| SAPD00427 | 6 | 314 |
| SAPD00330 | Un_random | 317 |
| 220_EP-PT | 7 | 319 |
| SAPD00394 | 16 | 319 |
| SAPD00676 | 8 | 320 |
| SAPD00960 | 11 | 327 |
| SAPD00370 | 17 | 330 |
| cDN11P0006I19 | 5 | 331 |
| 188_EP-PT | 6 | 333 |
| cDN08P0006E16 | 3 | 333 |
| SAPD00578 | 4 | 333 |
| cDN12P0006G10 | 13 | 339 |
| cDN03P0003D02 | Un_random | 340 |
| AJ291667 | 16 | 341 |
| cDN01P0005F03 | Un_random | 350 |
| SAPD00301 | 7 | 350 |
| SAPD00526 | 2 | 350 |
| SAPD01135 | Un_random | 352 |
| 606_IC-PT | 2 | 355 |
| cDN07P0006O14 | 5 | 357 |
| cDN11_CL42 | 13 | 358 |
| cDN13P0001J14 | 10 | 362 |
| AJ418660 | 14 | 367 |
| cDN05P0005J07 | 1 | 368 |
| cDN01P0004J02 | Un_random | 371 |
| 317_Sub23 | 17 | 376 |
| SAPD00880 | 1_random | 385 |
| cDN03P0002G13 | 10 | 387 |
| SAPD00062 | 1 | 397 |
| SAPD00612 | Un_random | 400 |
| SAPD00175 | 19 | 402 |
| cDN03P0005E06 | 6 | 411 |
| U30311 | 17 | 433 |
| SAPD01096 | 20 | 439 |
| SAPD00863 | 2 | 452 |
| SAPD01087 | 20 | 460 |
| cDN09P0005N14 | 11 | 462 |
| AF510084 | 16 | 480 |
| SAPD00110 | 5 | 500 |
| cDN07P0004A03 | 7 | 506 |
| cDN11P0004L03 | 6 | 517 |
| SAPD00470 | 1 | 518 |
| cDN03P0004L05 | 19 | 559 |
| SAPD01043 | Un_random | 574 |
| cDN06_CL213 | 1 | 578 |
| SAPD00958 | 1 | 627 |
| SAPD00935 | 18 | 641 |
| AF438176 | 12 | 712 |
| AF399824 | 5 or 13? | 764 |
| AF169368 | 12 | 844 |
| AF011223 | Un_random | 883 |
| AF184170 | 21?? | 1291 |
| AJ289717 | 7 | 1711 |
